# Genital mycobacteriosis caused by *Mycobacterium marinum* detected in two captive sharks by peptide nucleic acid–fluorescence in situ hybridization

**DOI:** 10.1111/jfd.13716

**Published:** 2022-09-21

**Authors:** Mari Inohana, Takeshi Komine, Yoshiaki Tanaka, Osamu Kurata, Shinpei Wada

**Affiliations:** ^1^ Laboratory of Aquatic Medicine, School of Veterinary Medicine, Faculty of Veterinary Medical Science Nippon Veterinary and Life Science University Musashino Japan; ^2^ Shimane Aquarium Hamada Japan

**Keywords:** elasmobranch, mycobacteriosis, *Mycobacterium marinum*, PNA‐FISH, zoonosis

## Abstract

*Mycobacterium marinum* is a prevalent nontuberculous mycobacterium (NTM)‐infecting teleosts. Conversely, little is known about mycobacteriosis in elasmobranchs, and *M. marinum* infection has never been reported from the subclass. This study investigated the histopathological characteristics and localization of this mycobacterium through molecular analysis of two captive sharks, a scalloped hammerhead *Sphyrna lewini* and a Japanese bullhead shark *Heterodontus japonicus*, exhibited in the same aquarium tank. We detected genital mycobacteriosis caused by *M. marinum* infection using molecular analyses, including polymerase chain reaction (PCR) and DNA sequencing targeting the 60 kDa heat‐shock protein gene (*hsp65*), and peptide nucleic acid–fluorescence in situ hybridization (PNA‐FISH) targeting the 16S rRNA gene. Both sharks showed granulomas in connective tissues of the gonads without central necrosis or surrounding fibrous capsules, which is unlike the typical mycobacterial granulomas seen in teleosts. This study reveals that elasmobranchs can be aquatic hosts of *M. marinum*. Because *M. marinum* is a representative waterborne NTM and a potential zoonotic agent, cautious and intensive research is needed to overcome a lack of data on the relationship between NTM and the aquatic environment in association with this subclass of Chondrichthyes.

## 1 INTRODUCTION

Nontuberculous mycobacterial infection is a chronic bacterial disease caused by *Mycobacterium* species other than *M. tuberculosis* complex and *M. leprae* (Jacobs, Stine et al., 2009). Nontuberculous mycobacteria (NTM) are gram‐positive, acid‐fast, non‐motile, rod‐shaped bacteria and are one of the most common causes of chronic disease in freshwater and marine fishes worldwide, especially in aquaria (Noga, 2010). Infections with NTM have significant impacts on cultured and wild food fishes and laboratory fish (Brocklebank et al., 2003; Rhodes et al., 2004; Whipps et al., 2012) and are associated with multiple symptoms, such as weight loss, skin ulceration and granulomas in the liver, kidney and spleen (Ferguson, 2006; Noga, 2010). Both freshwater and marine fishes are considered susceptible to NTM, most commonly *M. marinum*, *M. fortuitum* and *M. chelonae* (Gauthier & Rhodes, 2009). In addition to its impact on fish health, *M. marinum* is a potential zoonotic since it produces granulomatous lesions in human skin (Tomas et al., 2014), usually following exposure to infected fish surfaces (Jernigan & Farr, 2000) and is also a risk factor for pneumonia (Oh et al., 2018). In comparison with the wealth of information on piscine mycobacteriosis in teleosts, there is a remarkable paucity of reports about this disease in elasmobranchs.

Considering that water and biofilms are the natural habitats of *Mycobacterium* spp. (Pedley et al., 2004), accurate detection of causative pathogens in clinical cases of mycobacterial contamination in a fish‐rearing environment is a frequent problem. As such, there is a need to develop a tool that can localize gene expressions of *Mycobacterium* spp. using tissue sections from infected individuals. Fluorescence in situ hybridization (FISH) is an important candidate method that is based on the specific binding of nucleic acid probes to specific regions on targeted DNA in a tissue sample. Peptide nucleic acid (PNA) probes have been applied to the detection of mycobacteria using clinical samples from humans (Kim et al., 2015; Lefmann et al., 2006; Stender, Lund et al., 1999) because the relative hydrophobic character of PNA, as compared with DNA and RNA, allows better diffusion of the PNA probes through the cell wall of mycobacteria (Stender, Mollerup et al., 1999). However, nothing is known about FISH protocols using PNA probes to detect specific mycobacterial species in piscine mycobacteriosis.

The scalloped hammerhead *Sphyrna lewini* (Griffith & Smith) occurs circumglobally in warm‐temperate and tropical coastal seas (Compagno, 1984); it is one of the most threatened sharks and was listed as Critically Endangered (CR) by the IUCN Red List of Threatened Species in 2019 (Rigby et al., 2019). The Japanese bullhead shark *Heterodontus japonicus* Miklouho‐Maclay & Mcleay occurs in continental shelf waters of the northwestern Pacific, most commonly over rocky, kelp‐covered or sandy bottoms (Compagno, 2001). Both shark species have been successfully maintained in Japanese aquaria for exhibition and study.

Here, we present the first reported cases of *M. marinum* infection from two sharks that died in captivity. The infections were confirmed by peptide nucleic acid–fluorescence in situ hybridization (PNA‐FISH).

## 2 MATERIALS AND METHODS

### 2.1 Spontaneous mycobacteriosis and environmental samples

**FIGURE 1:**
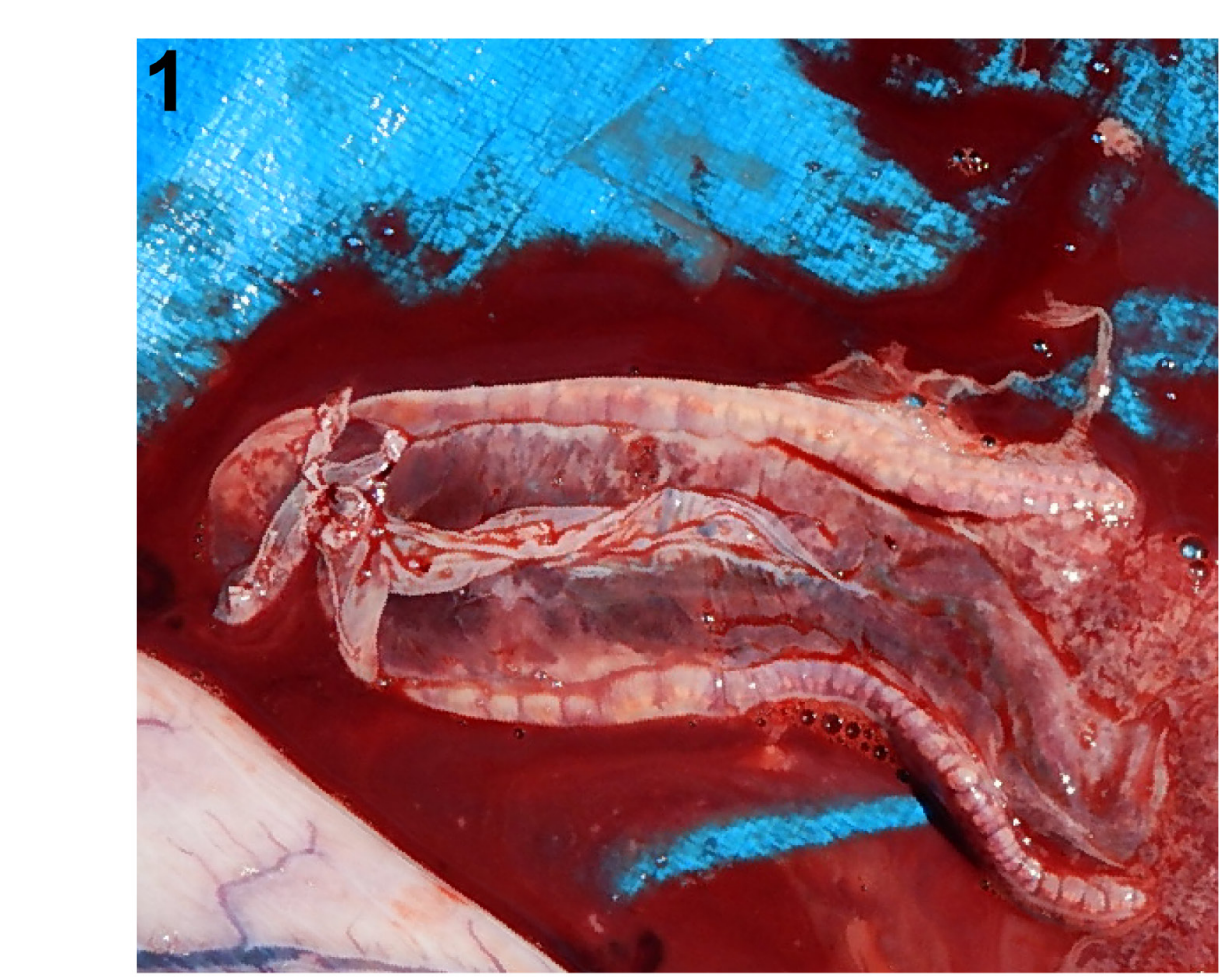
Testes and epigonal organ, scalloped hammerhead shark. Multifocal congestions in the bilateral testes and multifocal haemorrhages in the bilateral epigonal organ.

**FIGURE 2:**
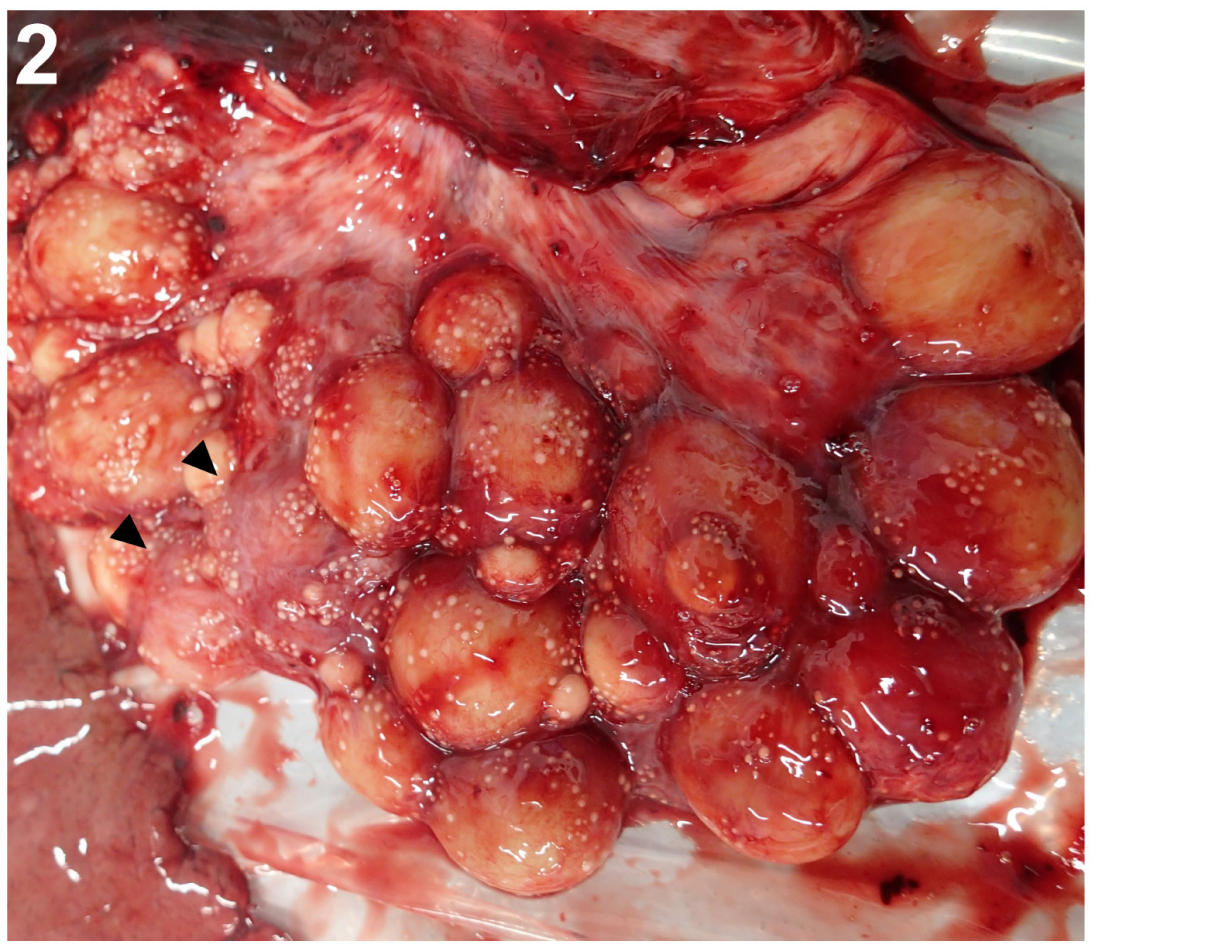
Ovary, Japanese bullhead shark. Oedema of the ovarian surface (arrowhead).

An approximately 10‐year‐old wild‐caught male scalloped hammerhead shark and a wild‐caught adult female Japanese bullhead shark of uncertain age were housed at the Shimane Aquarium in Shimane Prefecture, Japan. They were exhibited in a 1 million‐L tank exhibit along with other fish, including multiple teleost and elasmobranch species, and one sea turtle. Water pumped from the local sound (Sea of Japan), treated with pressure filtration, is used as the aquarium's water source. After 9 years and 9 months in captivity, the hammerhead shark presented anorexia for 1 month and was subsequently found dead in the tank. One month later, the bullhead shark was also found dead, but without any remarkable clinical signs. The captivity duration of this bullhead shark was unknown because Japanese bullhead sharks in the tank were not individually identified. Autopsies revealed marked hemocelom, multifocal congestions in the bilateral testes of the hammerhead shark and multifocal haemorrhages in the bilateral epigonal organ (Figure 1), while the bullhead shark displayed ovarian oedema (Figure 2). Apparent clinical and gross features of mycobacterial suspicious lesions were not detected in any of the remaining fish and the sea turtle in the tank.

To assess the microbial community distribution in the rearing environment, samples of water (1 L) and filtration sand, as well as swabs of biofilms from a wall and a water intake were provided. All samples were sent to the Laboratory of Aquatic Medicine at Nippon Veterinary and Life Science University in Tokyo.

### 2.2 Histopathological analysis

Samples from the brain, alimentary canal, liver, kidney, spleen, pancreas and reproductive organs were collected from both sharks, and samples from the epigonal organ, eyes and skin were collected from the hammerhead shark. The reproductive organs from the bullhead shark were frozen on site and then fixed in 10% neutral‐buffered formalin at our laboratory; the other remaining samples were fixed in 10% formalin on site. All samples were routinely processed to create paraffin‐embedded tissue blocks, sectioned at 2‐μm thickness, mounted on frosted glass slides, stained with haematoxylin and eosin (HE), Giemsa, Gram and Ziehl–Neelsen (ZN) stains and subjected to histopathological examination.

### 2.3 Nontuberculous mycobacteria (NTM) isolation

#### 2.3.1 Tissue samples

Isolation of the etiological agent of mycobacteriosis was attempted from frozen organs, including the reproductive organs, kidney, spleen and liver, in both cases, and the epigonal organ from the hammerhead shark. Pretreatments were performed based on four methods, as follows: (1) non‐treatment; (2) decontamination by hydrochloric acid (HCl) solution, at a final concentration of 0.5 N (Palomino & Portaels, 1998); or (3) decontamination by N‐acetyl‐l‐cysteine–sodium hydroxide (NALC‐NaOH) solution, based on diagnostic methods in a handbook by the (European Centre for Disease Prevention and Control, 2018), containing 2.0% NaOH and 0.5% NALC; or (4) dilution by Middlebrook 7H9 Broth. Thereafter, the samples were inoculated on BD™ Middlebrook 7H11 Agar supplemented with 10% OADC enrichment and 0.05% Tween 80 and 2% Ogawa medium (Kyokutou, Japan). These were allowed to incubate at 25 or 30°C for 8 weeks. At 28 and 56 days, a loopful of a visible colony was stained with ZN stain, and ZN‐positive colonies were purified.

#### 2.3.2 Environmental samples from the rearing tank

We performed NTM isolation from the tank water, the filtration sand, and the swabs of biofilm sampled from a wall and a water intake, following the previous methods of Thomson et al. (2008), Parashar et al. (2004) and Thomson et al. (2013), respectively, with small modifications. Following pretreatment and inoculation, the colonies were counted and purified as per the isolations of the tissue samples.

### 2.4 Molecular biological analyses

#### 2.4.1 Polymerase chain reaction (PCR)

The kidney, liver and spleen of both cases, and the testes and epigonal organ of the hammerhead shark were collected and fixed in ethanol. The ovary, uterus and shell gland of the bullhead shark were sampled and frozen. Mixed genomic DNA was extracted from the ethanol‐fixed or frozen samples, and the pellets of isolates were cultured from the tissues or environmental samples using a QIAamp DNA Mini Kit (Qiagen). The DNA was tested for the presence of mycobacterial DNA by PCR on the C1000TM Thermal Cycler (Bio‐Rad) using GoTaq® DNA Polymerase (Promega) and mycobacterial universal primers targeting the 16S ribosomal RNA (16S rRNA), 65 kDa heat‐shock protein (*hsp65*), the beta subunit of RNA polymerase (*rpo*B) and superoxide dismutase (*sod*A), through the cycles described by Fukano et al. (2017). The primer sets are listed in Table S1. Products were separated on a 2% agarose gel and the amplicon was purified using a NucleoSpin Gel and PCR Clean‐up Kit (Macherey‐Nagel).

The DNA showing a high percentage of nucleotide sequence identities to *M. marinum* was selected to confirm the presence of insertion sequences IS*2404* and IS*2606*, which relate to mycolactone‐producing mycobacteria (MPM), using the primer sets MU5–MU6 and PU4F–PU7Rbio for IS*2404*, and MU7–MU8 for IS*2606* (Table S1). The cycling conditions with the primer sets MU5–MU6 and MU7–MU8 are described by Stinear et al. (1999). The cycling condition with the primer set PU4F–PU7Rbio is described in Phillips et al. (2005), except that an initial hold step of 95°C for 10 min was used. The examination of genomic DNA was performed with the reference strains *M. marinum* ATCC BAA‐535 and *M. marinum* JCM 17638^T^ as negative controls, and *M. pseudoshottsii* JCM15466 as a positive control of both insertion sequences.

#### 2.4.2 DNA sequencing and phylogenetic analysis

Purified products were sequenced by the FASMAC Corporation using the specific primers described in Table S1. The resulting sequences were assembled and aligned using ClustalW in MEGA X software (Kumar et al., 2018). Sequence identities were established using the NCBI Basic Local Alignment Search Tool (BLAST) software (http://www.ncbi.nlm.nih.gov/blast/Blst.cgi). The phylogenetic tree of the *hsp65* sequences was reconstructed using the neighbour‐joining method with Kimura's 2‐parameter model, implemented in MEGA X software and evaluated further in a bootstrap analysis of 1000 replicates.

### 2.5 Peptide nucleic acid (PNA)–Fluorescence in situ hybridization (FISH)

#### 2.5.1 Bacterial and tissue samples

To estimate the hybridizability and specificity of the designed PNA probes and to validate the PNA‐FISH procedure, mycobacterial isolates from the tissues of the current clinical cases and the reference strains *M. marinum* ATCC BAA‐535 and *M. marinum* JCM 17638^T^ were used. To detect pathogens in the shark tissues, paraffin‐embedded tissue samples of the current cases, including the testes and epigonal organ of the hammerhead shark, and the ovary, uterus and shell gland of the bullhead shark, were used. As a negative tissue control, the ovary of another hammerhead shark, in which mycobacterial infection was not detected by ZN staining and by PCR assay, was also tested.

#### 2.5.2 PNA probes

With the genome and partial sequence database using BLAST, and the results of sequence analyses of DNA extracted from the present samples, we designed four PNA probes (Table 2), each of which was designed to detect the targeted 16S rRNA of the specific NTM species among the four candidate etiological agents detected as the genomic evidence from the tissue samples and/or the isolated strains in this study, including *M. marinum*, *Mycolicibacterium llatzerense* (NJB1901), *M. hodleri* (NJB19061) and *M. obuense* (NJB19062). Each probe was designed not to cross‐hybridize with the non‐targeted genomic sequences of the other NTM candidates, and therefore, it acted as a negative control for the other three NTM species like a sense probe. The probes were chosen with regard to purine content, avoiding hairpin formations and inverted repeats. The N‐terminus of all probes were labelled with cyanine 3 (Cy3) fluorescent dye. All designed probes were custom‐synthesized by PANAGENE Inc.

#### 2.5.3 FISH procedure

The preparation of tissue sections and bacterial samples for FISH was performed as described in Lehtola et al. (2006) and Lefmann et al. (2006), respectively. Both samples were preheated to the hybridization temperature of 60°C. The hybridization protocol was performed as previously reported in Lefmann et al. (2006) by applying a 50‐μl aliquot of hybridization solution containing a fluorescent probe with a final concentration of 1.5 mol/L to each sample and then incubating the samples at 60°C for hybridization and washing. Following the hybridization procedure, slides were stained for 10 min in 600 μM 4′,6‐diamidino‐2‐phenylindole (DAPI; Life Technologies), washed in DW for 5 min, air dried and then mounted with 1 drop of Vectashield (Vector Laboratories). Slides were covered with a glass coverslip, and the edges were sealed with clear nail polish. As a negative control, the hybridization procedure was performed without a probe added.

#### 2.5.4 Microscopy

Microscopy was performed with a fluorescence microscope (Keyence BZ‐X700). TRITC OP‐87764 and DAPI OP‐87762 filter sets (Keyence) were used to analyse the Cy3 and DAPI signals, respectively. Digital images were captured under the fluorescence microscope, and image analysis was performed with BZ‐H3 software (Keyence).

## 3 RESULTS

### 3.1 Histopathological analysis

**FIGURE 3:**
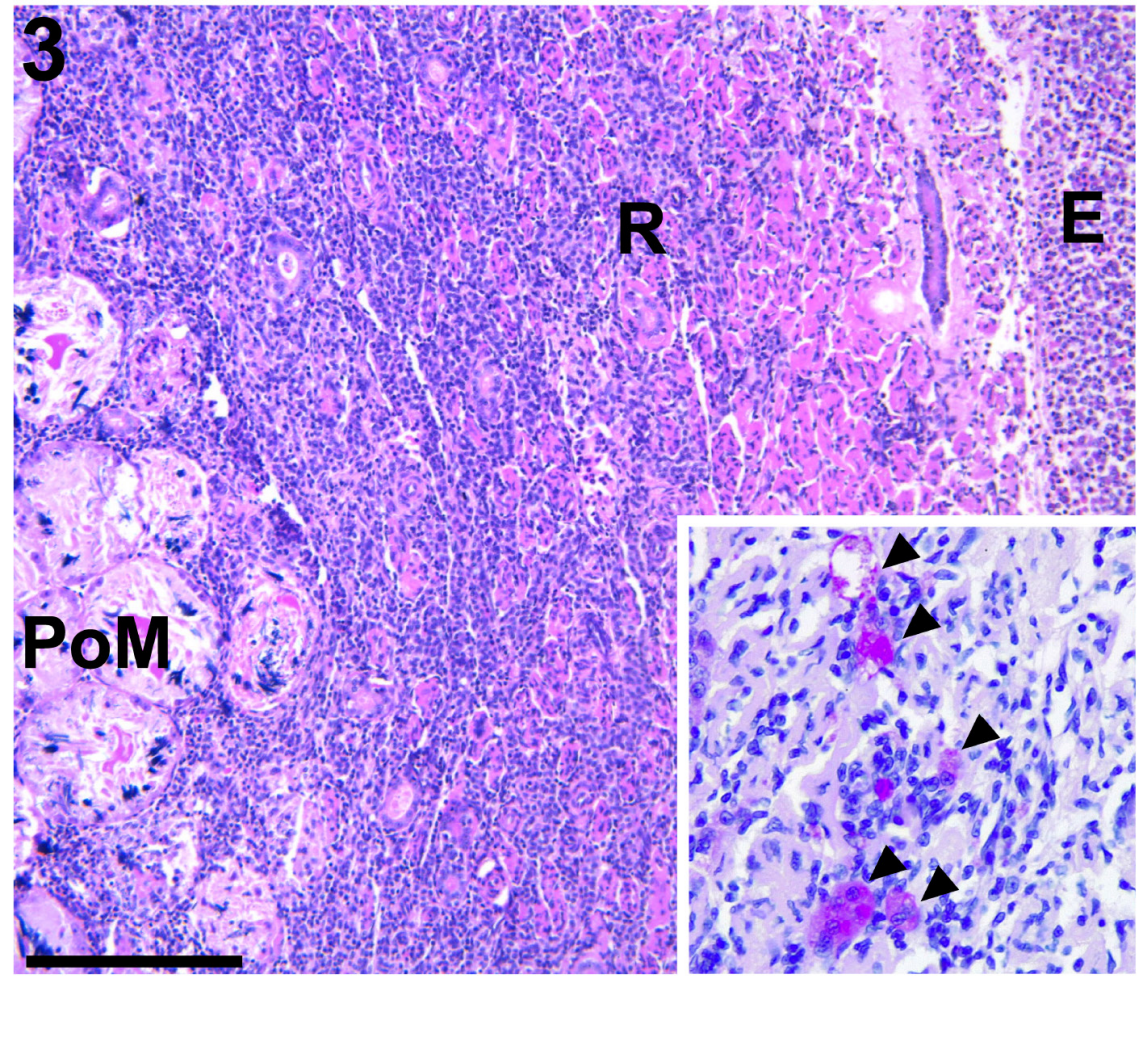
Testis, scalloped hammerhead shark. In the resorption zone, foamy macrophages infiltrated into the connective tissue mixing with large numbers of infiltrating lymphocytes, shown using HE staining. E, epigonal organ; PoM, postmeiotic zone; R, resorption zone. Bar = 200 μm. Inset: Numerous acid‐fast bacilli in cytoplasm of the macrophages (arrowhead), visible with Ziehl–Neelsen stain.

In both testes of the hammerhead shark, foamy macrophages infiltrated into the serosal connective tissues in the resorption zone mixing with large numbers of infiltrating lymphocytes (Figure 3). Numerous acid‐fast bacilli (AFB) were observed within the macrophages comprising the granulomatous inflammation by using ZN stain (Figure 3, inset), but the AFB did not infiltrate into the epigonal organ. Thus, the testes of the hammerhead shark were morphologically diagnosed as: ‘granulomatous serositis with intralesional AFB’.

**FIGURE 4:**
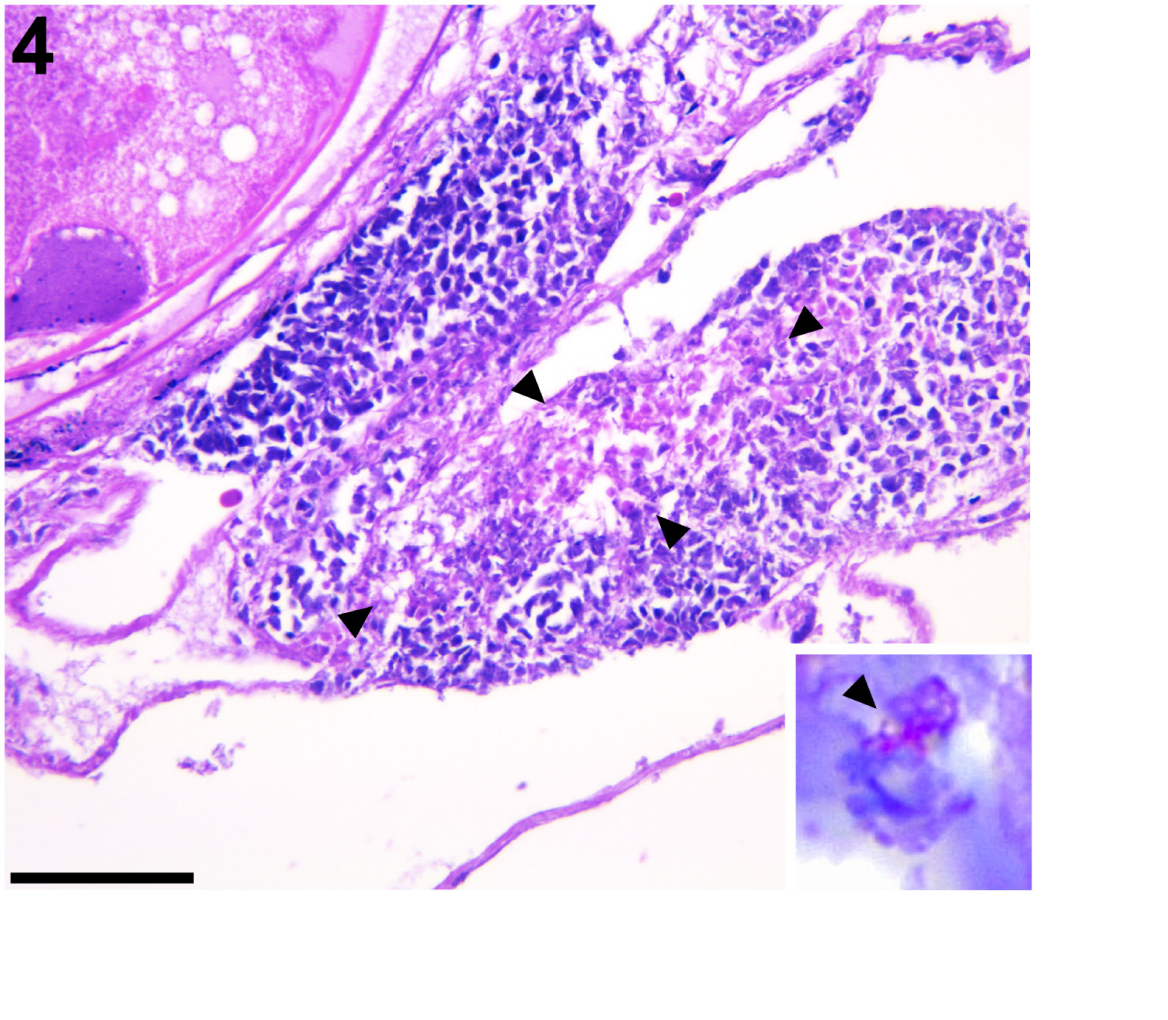
Ovary, Japanese bullhead shark. Infiltration and accumulation of macrophage‐like cells with apparent, foamy cytoplasm in the ovarian connective tissue stroma (surrounded by arrowheads), shown using HE staining. Bar = 50 μm. Inset: A part of the accumulation showed acid‐fast bacilli within cytoplasm of the macrophage‐like cell (arrowhead), visible with Ziehl–Neelsen stain.

Although it was somewhat difficult to distinguish inflammation cells since the sample was frozen after sampling, macrophage‐like cells with foamy cytoplasm infiltrated and accumulated in the ovarian connective tissue stroma of the bullhead shark (Figure 4). ZN staining revealed that a part of the accumulation showed numerous AFB within macrophage‐like cells (Figure 4, inset). Thus, the morphological diagnosis of the ovary of the bullhead shark was: ‘granulomatous capsulitis with intralesional AFB’.

In the hammerhead shark, in addition to mycobacteriosis, we diagnosed panophthalmitis, corneal trephination, enteritis, dermatitis, focal lymphocytic meningitis and suspected collagenofibrotic glomerulopathy. In the bullhead shark, we additionally diagnosed focal lymphocytic gastritis, focal lymphocytic interstitial nephritis, pancreatitis and necrotic splenitis. However, in both cases, no apparent pathological features of mycobacterial suspicious lesions were detected other than in the testes or ovaries.

### 3.2 NTM isolations

#### 3.2.1 Tissue sampling

In total, three AFB strains were isolated from the testis of the hammerhead shark (NJB1901) and the ovary of the bullhead shark (NJB19061 and NJB19062). NJB1901 was cultured using Middlebrook 7H11 agar without pretreatment; on day 21 after inoculation at 30°C, it formed a smooth, white colony. NJB19061 and NJB19062 were cultured using the same agar without pretreatments; on day 14 after inoculation at 25 and 30°C, respectively, each formed a smooth, yellow colony. All purified colonies of the three isolates showed rapid growth on day 3, with Middlebrook 7H11 agar, at 25°C (NJB19061) or 30°C (NJB1901, NJB19062).

#### 3.2.2 Environmental samples from the rearing tank

Seven isolates of mycobacteria were cultured from the environmental samples. The colony characteristics of the isolates are presented in Table S2.

### 3.3 Molecular biological analyses

#### 3.3.1 Housekeeping genes of mycobacterium

Amplicons of the appropriate molecular weight were produced by PCR of the DNA extracted from four parts of the bilateral testes (I‐a and I‐b [left testis], I‐c and I‐d [right testis]) and from the ovary, using primers to amplify the *hsp65* gene, and from one part of the left testis (testis II) using primers to amplify the 16S rRNA, *hsp65*, *rpo*B and *sod*A genes. Sequence analyses indicated that *M. marinum* was the most probable candidate bacterial species detected in four portions of the testes (I‐a, I‐b, I‐c and I‐d) of the hammerhead shark, and in the ovary of the bullhead shark, based on genomic evidence, whereas *M. llatzerense* was the most probable candidate species in testis II of the hammerhead shark, based on the high degree of molecular homology (Table 1).

**TABLE 1:** Nucleotide sequence homology (%) of PCR products from testis tissues of the scalloped hammerhead shark and ovary tissue of the Japanese bullhead shark

Gene	16S rRNA	*hsp65* (401 bp)	*rpo*B (315 bp)	*sod*A (419 bp)
Tissues				
Testis I^a^	NA^b^	*Mycobacterium marinum* (100)	NA^b^	NA^b^
Testis II	*Mycolicibacterium llatzerense* (100)^c^	*Mycolicibacterium llatzerense* (99.5)	*Mycolicibacterium llatzerense* (99.6)	*Mycolicibacterium llatzerense* (98.8)
Ovary	NA^b^	*Mycobacterium marinum* (100)	NA^b^	NA^b^
Isolates				
NJB1901	*Mycolicibacterium llatzerense* (100)^c^	*Mycolicibacterium llatzerense* (99.5)	*Mycolicibacterium llatzerense* (99.6)	*Mycolicibacterium llatzerense* (98.8)
NJB19061	*Mycolicibacterium hodleri* (100)^c^	*Mycolicibacterium hodleri* (98.0)	*Mycolicibacterium hodleri* (99.6)	*Mycolicibacterium hodleri* (96.9)
NJB19062	*Mycolicibacterium obuense* (99.8)^c^	*Mycolicibacterium obuense* (100)	*Mycolicibacterium obuense* (100)	*Mycolicibacterium chelonae* (99.7)

*Note*: The table lists mycobacterial species of which the sequence of PCR products of DNA extracted from the samples show the highest homology with 100% query coverage to each housekeeping gene.

Abbreviations: 16S rRNA, 16S ribosomal RNA; *hsp65*, 65 kDa heat‐shock protein; NA, not applicable; *rpo*B, the beta subunit of RNA polymerase; *sod*A, superoxide dismutase.

^a^ Four portions of the testes (I‐a, I‐b [left testis], I‐c and I‐d [right testis]) showed the same results.

^b^ The sequencing analysis was not performed because the results of the PCR were negative.

^c^ The length of partial 16S rRNA gene sequences of testis II, NJB1901, NJB19061 and NJB19062 were 1292 bp, 1438 bp, 1444 bp and 1439 bp, respectively.

**FIGURE 5:**
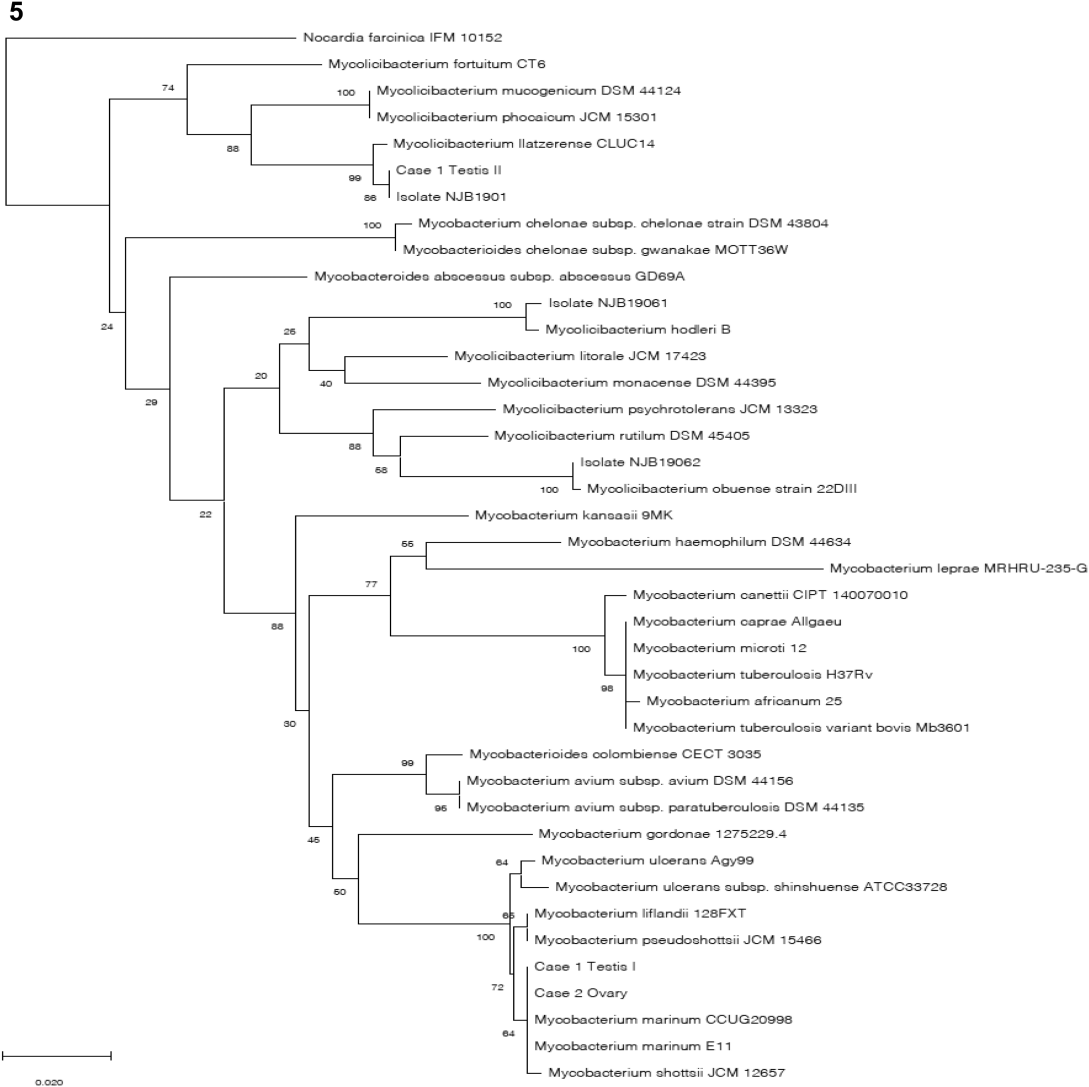
Neighbour‐joining tree generated from a concatenated 401‐bp sequence of the *hsp65* gene from two parts of the testes of the scalloped hammerhead shark (Case 1: Testis I, testis II), the ovary of the Japanese bullhead shark (Case 2: Ovary), and the three isolates cultured from the sampled tissues (NJB1901, NJB19061, NJB19062) with Kimura's two‐parameter distance correction model. Bootstrap values are indicated at nodes as a percentage of 1000 replicates.

Polymerase chain reaction of DNA extracted from the isolates produced amplicons of the appropriate molecular weights using primers to amplify the 16S rRNA, *hsp65*, *rpo*B and *sod*A genes. The results of sequence analyses of the isolates showed that NJB1901, NJB19061 and NJB19062 were identical with *M. llatzerense*, *M. hodleri* and *M. obuense*, respectively (Table 1). The nucleotide sequences of the isolates from the environmental samples were identical to some NTM species other than those detected in the tissue samples (Table S2). The nucleotide sequence data are available in the DDBJ/EMBL/GenBank databases under accession nos. LC713220–LC713257. Figure 5 shows the results of the phylogenetic analysis with the isolates and the genomic evidence from tissue samples using a 401‐bp sequence of the *hsp65* gene.

#### 3.3.2 IS2404 and IS2606

**FIGURE 6:**
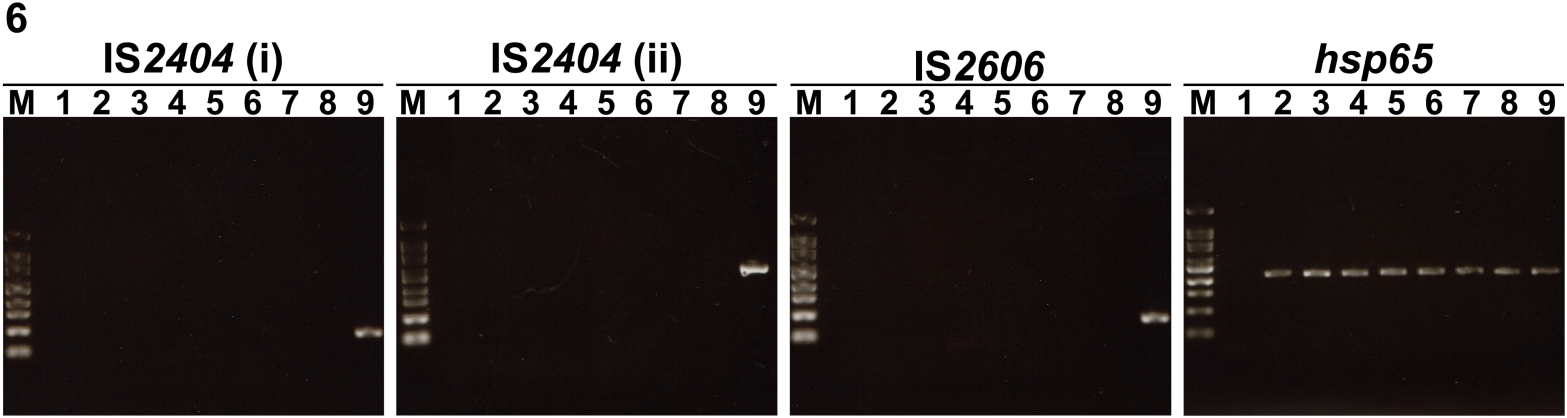
Results of gel electrophoresis of PCR products to amplify the insertion sequence IS*2404* with the primer sets PU4F–PU7Rbio (i) or MU5–MU6 (ii), the insertion sequence IS*2606* with the primer set MU7–MU8, or the *hsp65* gene with the primer set TB11–TB12 in 2% agarose gel. lane 1, water; lane 2, testis I‐a; lane 3, testis I‐b; lane 4, testis I‐c; lane 5, testis I‐d; lane 6, ovary; lane 7, *Mycobacterium marinum* ATCC BAA‐535; lane 8, *M. marinum* JCM17638^T^; lane 9, *M. pseudoshottsii* JCM15466; lane M, 100‐bp DNA Ladder RTU (GeneDireX Inc.)

No apparent bands were detected for the insertion sequences IS*2404* and IS*2606* with the DNA extracted from testis I of the hammerhead shark and from the ovary of the bullhead shark, or from the negative controls (*M. marinum* ATCC BAA535 and *M. marinum* JCM17638^T^), while apparent bands were observed with DNA extracted from the positive control (*M. pseudoshottsii* JCM15466) (Figure 6).

### 3.4 PNA‐FISH

#### 3.4.1 Probe design and evaluation

**FIGURES 7–12:**
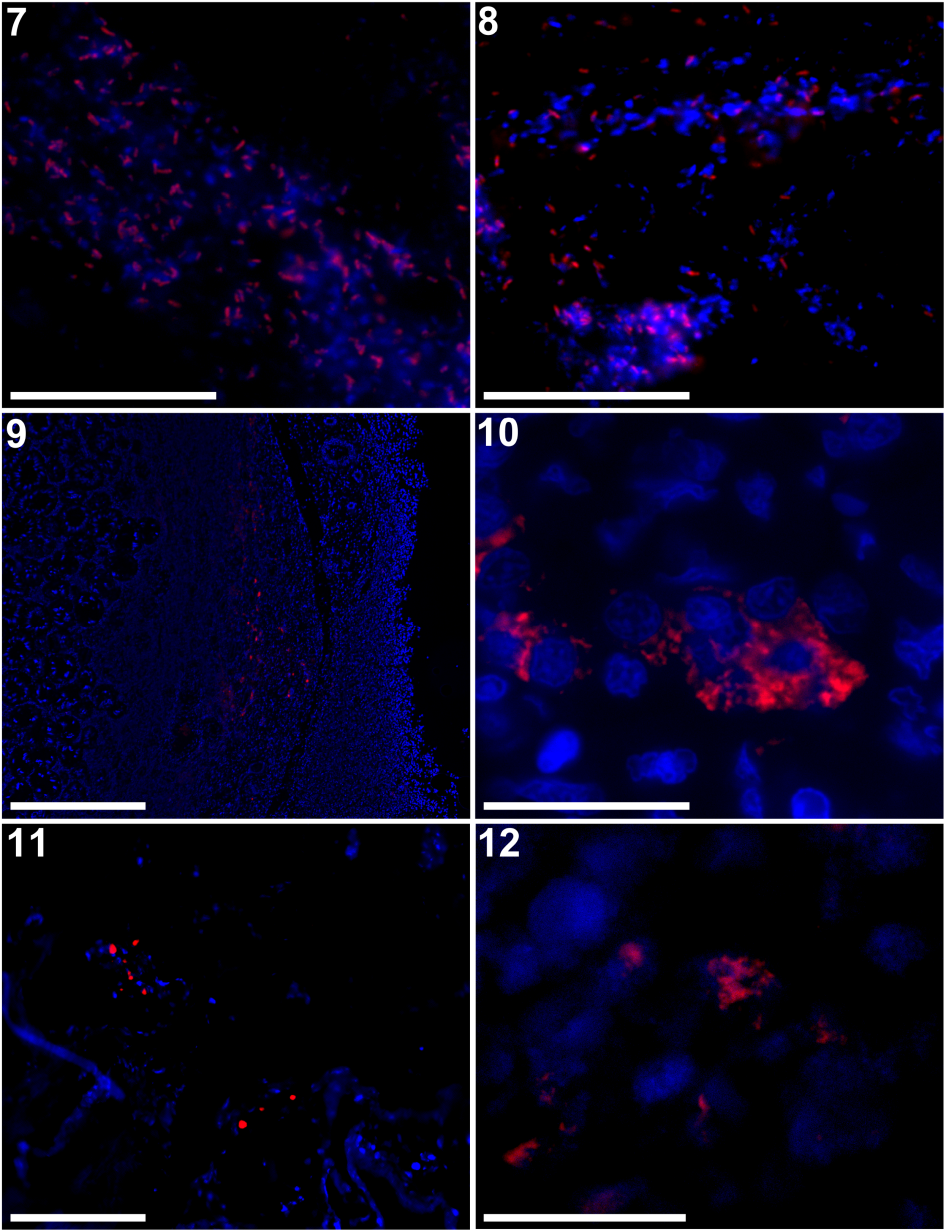
Visualization by fluorescence in situ hybridization (FISH), superimposed with MM probe (red) and DAPI stain (blue) of Mycobacterium marinum JCM17638T (Figure 7) and of M. marinum ATCC BAA‐535 (Figure 8). Rod‐ or dot‐shaped red signals were apparent at high magnification. Bar = 20 μm. (Figures 9 and 10) Testis, scalloped hammerhead shark. Visualization by FISH superimposed with MM probe (red) and DAPI stain (blue) (Figure 9). The red signals were located in the resorption zone, where acid‐fast bacilli were evident from Ziehl–Neelsen staining. Bar = 500 μm (Figure 10). Rod‐ or dot‐shaped red signals beside the nuclei, blue signal, at high magnification. Bar = 20 μm. (Figures 11 and 12) Ovary, Japanese bullhead shark. Visualization by FISH superimposed with the MM probe (red) and DAPI stain (blue) (Figure 11). The rod signals were located at the connective tissue near follicles. Bar = 500 μm (Figure 12). Rod‐ or dot‐shaped red signals beside the nuclei, blue signal. Bar = 20 μm.

Probes of 15 bp each were designed to match 100% with the 16S rRNA of each target *Mycobacterium* species (Table 2). FISH with probes MM, ML, MH and MO resulted in the correct identification of the *M. marinum* reference strain, and the NJB1901, NJB19061 and NJB19062 isolates, respectively. The results of FISH with strains *M. marinum* JCM 17638^T^ and ATCC BAA‐535 using probe MM are shown in Figures 7 and 8; all results are compiled in Figure S1. Fixed bacterial cells showed no fluorescence in the PNA‐FISH experiments lacking a probe (negative control). All targeted mycobacteria were stained by each specific probe and were visible as single cells or clusters.

**TABLE 2:** Sequences of the peptide nucleic acid (PNA) probes and the complementary 16S rRNA sequences of the target mycobacterial species and isolates

Probe	Sequence	Target species/isolates^b^
MM	TCC TGG TGC CCT AAG‐OO‐Cy3 (C‐N)	
	AGG ACC ACG GGA TTC^a^ (5′‐3′)	*Mycobacterium marinum*
ML	TAC TGG TGC GAG AAG‐OO‐Cy3 (C‐N)	
	ATG ACC ACG CTC TTC (5′‐3′)	NJB1901, *Mycolicibacterium llatzerense*
MH	TAC TGG AAC CCT ACG‐OO‐Cy3 (C‐N)	
	ATG ACC TTG GGA TGC (5′‐3′)	NJB19061, *Mycolicibacterium hodleri*
MO	TCC TGG TGC GCG AAG‐OO‐Cy3 (C‐N)	
	AGG ACC ACG CGC TTC (5′‐3′)	NJB19062, *Mycolicibacterium obuense*

Abbreviation: 16S rRNA, 16S ribosomal RNA.

^a^ The complementary 16S rRNA sequences of the target mycobacterial species and isolates.

^b^ The mycobacterial species and isolates, which each probe targets.

#### 3.4.2 Mycobacteria in tissue sections

After probe evaluation using FISH for the type strains and the isolates, PNA‐FISH was used to visualize mycobacteria in fixed tissue sections. Testes sections of the hammerhead shark infected with *Mycobacterium* spp. were examined with probe MM, showing the distribution of red signals at the edge of the testes adjacent to the epigonal organ and in the muscular layer between the testis and epigonal organ (Figure 9), where macrophages with intracellular AFB were found aggregated, as revealed by ZN stain in the histopathological examination. At high magnification, rod‐ or dot‐shaped red signals were scattered and accumulated immediately adjacent to nuclei, which were stained by DAPI as blue signals (Figure 10). Ovary sections of the bullhead shark were stained with probe MM, demonstrating the distribution of red signals within the area of macrophage‐like cell aggregates with intracellular AFB, likewise revealed by ZN stain (Figure 11). High magnification of the ovary section showed the accumulations of rod‐ or dot‐shaped red signals, as seen in the testis section (Figure 12).

Conversely, the PNA probes designed for other candidate NTM (i.e., ML, MH and MO) did not show positive signals indicating the presence of the targeted NTM in the tissue sections of both samples. In the negative controls, no signal was detected in the tissue sections examined (Figure S2). Also, no signal was determined in other tissue sections, including the epigonal organ of the hammerhead, and the shell gland and uterus of the bullhead shark (data not shown).

## 4 DISCUSSION

In the present two cases involving a scalloped hammerhead shark and a Japanese bullhead shark that had been maintained in a public display aquarium, the sequence analyses of the extracted DNA from testis I and ovary tissue sections using the *hsp65* gene detected genomic evidence of nontuberculous mycobacteria (NTM) infection, with 100% nucleotide sequence identity to *M. marinum*. The nucleotide sequence of *hsp65* gene of *M. marinum* shows a relatively high rate of similarity with mycolactone‐producing mycobacteria (MPM), such as *M. pseudoshottsii*. However, in most *M. marinum* strains reported previously, the insertion sequences IS*2404* and IS*2606* relating to mycolactone were not detected by PCR, unlike detection in MPM (Rhodes et al., 2005; Stinear et al., 1999; Trott et al., 2004). Therefore, PCR analysis targeting IS*2404* and IS*2606* was used to distinguish most types of strains of *M. marinum* from MPM based on the absence of both these sequences.

The present study found that the *hsp65* gene was detected in DNA extracted from testis I and the ovary, but insertion sequences IS*2404* and IS*2606* were not detected in those tissues using primer sets that had been previously applied to clinical tissue samples and/or formalin‐fixed paraffin‐embedded samples (Jacobs, Howard et al., 2009; Phillips et al., 2005; Sakyi et al., 2016), as observed with the reference *M. marinum* strains examined. These features indicated that the DNA extracted from testis I and the ovary could be identified as *M. marinum*.

The phylogenetic analysis using the 401‐bp sequence of the *hsp65* gene also supported this identification, because these nucleotide samples (Figure 5: Case I Testis I, and Case 2 Ovary) were classified into the same cluster in which the other *M. marinum* reference strains, such as *M. marinum* CCUG20998 and E11, were included.

In this study, PNA‐FISH with MM probe revealed the existence of *M. marinum* by detecting its partial sequence in tissues of the reproductive organs in clinical cases in two shark species. Thus, the current study demonstrates that PNA‐FISH is useful for the diagnosis of mycobacterial infection in fish and in humans (Kim et al., 2015; Lefmann et al., 2006; Stender, Lund et al., 1999). From these overall results, we conclude that the current cases could be diagnosed as mycobacteriosis, with *M. marinum* as the likely pathogen, and NJB1901, NJB19061 and NJB19062 might be contaminating environmental microorganisms.

This study is the first to describe cases of *M. marinum* infection in an elasmobranch.

Mycobacteriosis cases in this subclass have rarely been reported (Anderson et al., 2012). A few examples to date include detections of infection by *M. chelonae* of the skin of a captive yellow stingray *Urobatis jamaicensis* (Cuviier) (Clarke et al., 2013), infection by *M. avium* of the oral cavity, spleen and surface of the claspers of a captive epaulette shark *Hemiscyllium ocellatum* (Bonnaterre) (Janse & Kik, 2012), infection by *M. chelonae* of the spleen of an Atlantic guitarfish *Pseudobatos lentiginosus* (Garman) (=*Rhinobatos lentiginosus*) (Anderson et al., 2012) and infection by *M. chelonae* of the gills, heart rectal gland, mesentery or liver in the case of two Atlantic guitarfish with septicaemia (Tuxbury et al., 2017). However, no clinical case of *M. marinum* infection has been identified previously. One study describing serological evidence of mycobacteriosis did suggest the possibility of *M. marinum* infection in sharks by measuring a bacteria‐specific IgM antibody in the Atlantic sharpnose shark *Rhizoprionodon terraenovae* (Richardson) (Karsten & Rice, 2006).

*Mycobacterium marinum* is a representative NTM agent in aquatic animals; it is considered an important candidate infectious agent of aquatic zoonosis because *M. marinum* has been isolated from human lesions, including dermatitis (Tomas et al., 2014), following exposure to contaminated aquatic materials (Jernigan & Farr, 2000), and is implicated in pneumonia as well (Oh et al., 2018). Based on these features it is vital to know the prevalence and distribution of NTM in aquatic environments and organisms, in accordance with the ‘One Health’ approach to control NTM infections in humans (Thirunavukkarasu et al., 2017). However, based on a paucity of research, the susceptibility of elasmobranchs to *M. marinum* infection and the potential for zoonotic transmission from an elasmobranch species have not yet been shown. Nevertheless, the present results prove that an elasmobranch species can be a host of waterborne *M. marinum* and indicate that elasmobranchs, like teleosts, might act as a reservoir of *M. marinum* infection in humans, especially immunocompromised individuals (Streit et al., 2006).

The progress of granuloma pathology is usually dependent on the duration and amount of infecting bacteria (Swaim et al., 2006) and is influenced by other factors, such as differences among host species (Antuofermo et al., 2017; Swaim et al., 2006) and the bacterial strain (van der Sar et al., 2004). Even so, mycobacteriosis in most teleosts is characterized by caseating granulomas (Noga, 2010). By contrast, the lesions in the reproductive organs of both cases of the sharks in our study showed granulomatous inflammation composed of loose aggregates of macrophages without central necrosis, capsules composed of fibrous tissue or epithelioid tissue and infiltrations of giant cells. Unlike the typical granulomas seen in teleosts, the histopathological features observed in the reproductive organs of the current cases might make the histopathological diagnosis of mycobacteriosis in an elasmobranch quite difficult.

In teleost fishes, the ovary and testis are not major organs involved in mycobacterial infection (Novotny et al., 2010), though have been described in rare cases, including spontaneous and experimental infections of *Mycobacterium* spp. in association with granulomatous inflammation (Broussard & Ennis, 2007; Whipps et al., 2008). In elasmobranchs, although clinical reproductive abnormalities (Anderson et al., 2012) and yolk coelomitis (Tuxbury et al., 2017) were reported in guitarfishes with mycobacterial infection, the involvement of some mycobacterial species in these abnormalities of the reproductive system was not determined, because no AFB were detected in the lesions of those cases. The presence of intralesional *M. marinum* in the reproductive organs in the current cases clarified that mycobacteria could cause gonadal lesions in elasmobranchs as in teleosts.

In conclusion, our study indicates that mycobacteriosis can be suspected in elasmobranchs even without typical caseating granulomas and it should be added to potential zoonotic diseases in aquaria housing sharks.

## CONFLICT OF INTEREST

The authors have no conflicts of interest to declare.

## Supporting information


Appendix S1

## Data Availability

The datasets generated during and/or analysed during the current study are available from the corresponding author upon reasonable request.
