# Supplementary material for: Genital mycobacteriosis caused by Mycobacterium marinum detected in two captive sharks by peptide nucleic acid–fluorescence in situ hybridization
Source: J Fish Dis. 2022 Sep 21;46(1):47–59. doi: 10.1111/jfd.13716 (PMC10087912; doi:10.1111/jfd.13716)
Supplement: Supplementary file 1 — Appendix S1 [file JFD-46-47-s001.docx]

**Supplementary Table S1**

**Sequences of primer sets used in the PCR and DNA sequencing analysis**

Genes Primers Sequences (5′-3′)

Housekeeping genes

16S rRNA 8F AGAGTTTGATCCTGGCTCAG

1046R TGCACACAGGCCACAAGGGA

830F GTGTGGGTTTCCTTCCTTGG

1542R AAGGAGGTGATCCAGCCGCA

*hsp65*  TB11 ACCAACGATGGTGTGTCCAT

TB12 CTTGTCGAACCGCATACCCT

*rpo*B MF CGACCACTTCGGCAACCG

MR TCGATCGGGCACATCCGG

*sod*A sodF ACATCTCGGGTCAGATCAACGAGC

sodR GACGTTCTTGTACTGCAGGTA

Insertion sequence

IS*2404* MU5 AGCGACCCCAGTGGATTGGT

MU6 CGGTGATCAAGCGTTCACGA

PU4F GCGCAGATCAACTTCGCGGT

PU7Rbio GCCCGATTGGTGCTCGGTCA

IS*2606* MU7 GGCCTGGCGGATTGCTCAAGG

MU8 CGTAGATGTGGGCGAAATGG

Abbreviations: 16S rRNA, 16S ribosome RNA; *hsp65*, 60 kDa heat-shock protein; *rpo*B, the β subunit

of bacterial RNA polymerase; *sod*A, superoxide dismutase.

**Supplementary Table S2**

**Characteristics of the colonies isolated from the environmental samples taken from the affected display tank housing the infected elasmobranchs**

Isolates Resources Treatment Colony morphological features Incubation period *Mycobacterium* sp. ^†^

for culture Surface Color (day)

NJB1901-2501 Filter sand HCl Smooth white 3 *M. fortuitum* complex

NJB1901-2504 Filter sand NaOH Smooth white 3 *M. fortuitum* complex

NJB1901-3001 Filter sand HCl Smooth white 5 *M. fortuitum* complex

NJB1901-3002 Filter sand HCl Smooth white 5 *M. chelonae*

NJB1901-3003 Filter sand HCl Smooth white 5 *M. fortuitum* complex

NJB1901-3004 Filter sand NaOH Smooth white 5 *M. fortuitum* complex

NJB1901-3000 Tank water dilution Smooth orange 5 *M. fortuitum* complex

^†^Shown are mycobacterial species of which sequencing of the PCR product of DNA extracted from each isolate demonstrated the highest nucleotide sequence identities with 100% query coverage

to the 16S rRNA, *hsp65*, *rpo*B and *sod*A genes.

Abbreviations: HCl, hydrochloric acid; NaOH, sodium hydroxide.


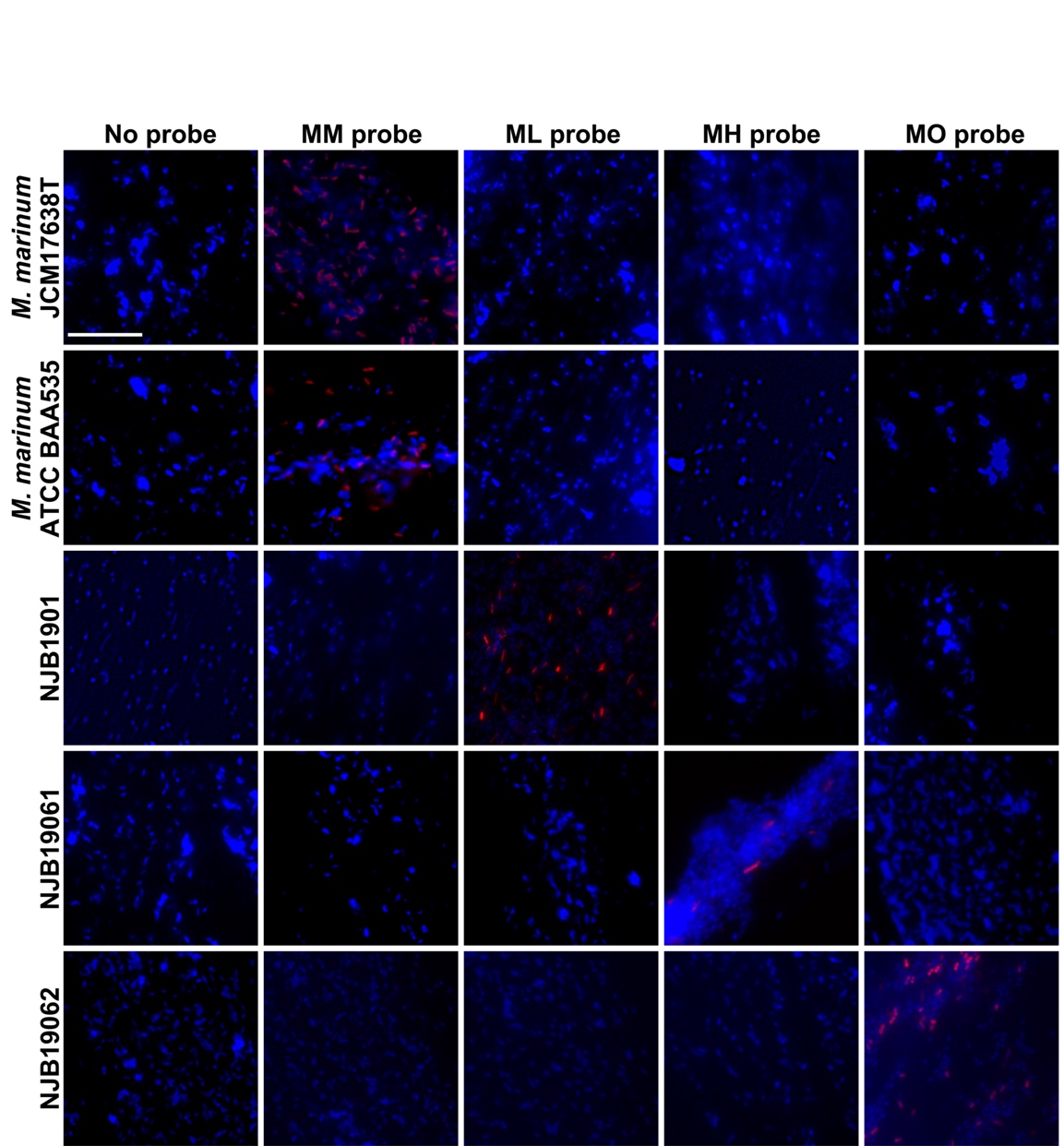


**Supplementary Figure S1**

Visualization by fluorescence *in situ* hybridization (FISH) superimposed with no probe, or probe MM, ML, MH or MO (red) and DAPI stain (blue) of the type strains (*Mycobacterium marinum* JCM17638^T^ and ATCC BAA-535) and the isolates (NJB1901, NJB19061, NJB19062). Fixed bacterial cells showed no fluorescence in the FISH experiments without a peptide nucleic acid (PNA) probe as a negative control. Only targeted mycobacteria were stained by each specific probe and visible as red single cells. Bar = 10 µm.


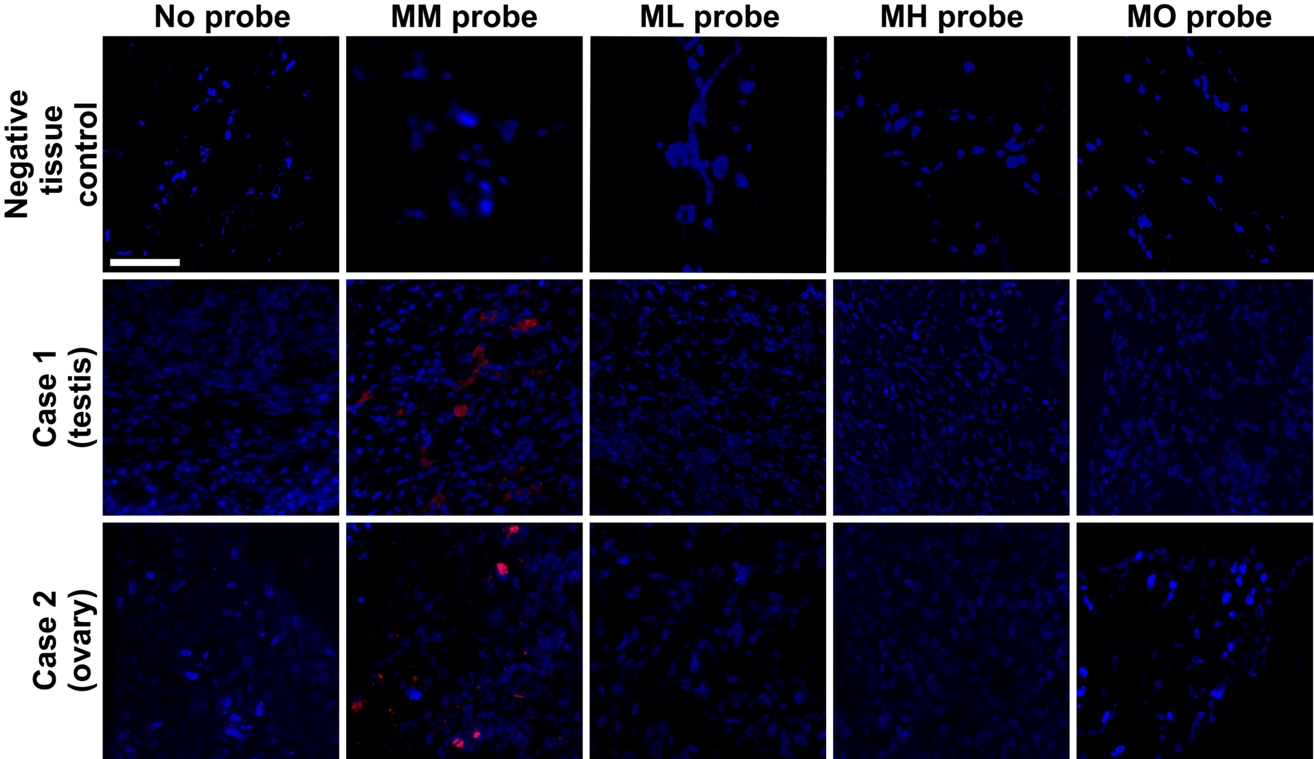


**Supplementary Figure S2**

Visualization by fluorescence *in situ* hybridization (FISH) superimposed with no probe, or probe MM, ML, MH or MO prove (red) and DAPI stain (blue) in the clinical case of the scalloped hammerhead shark (Case 1) and the Japanese bullhead shark (Case 2), and, in the negative tissue control, the ovary of another hammerhead shark. The red signals were besides nuclei (blue signals). Bar = 50 µm.
